# Host or the Hosted? Effects of Non-Nutritive Sweeteners on Intestinal and Microbial Mechanisms of Glycemic Control

**DOI:** 10.3390/nu16213628

**Published:** 2024-10-25

**Authors:** Braden D. Rose, Nektaria Pezos, Jocelyn M. Choo, Tongzhi Wu, Geraint B. Rogers, Kerry L. Ivey, Christopher K. Rayner, Richard L. Young

**Affiliations:** 1 Intestinal Sensing Group, The University of Adelaide, Adelaide, SA 5005, Australia; 2 Adelaide Medical School, The University of Adelaide, Adelaide, SA 5005, Australia; 3 Centre of Research Excellence in Translating Nutritional Science to Good Health, The University of Adelaide, Adelaide, SA 5005, Australia; 4 Diabetes, Nutrition & Gut Health, South Australian Health & Medical Research Institute (SAHMRI), Adelaide, SA 5000, Australia; 5 Microbiome and Host Health, Lifelong Health, South Australian Health & Medical Research Institute (SAHMRI), Adelaide, SA 5000, Australia; 6 Infection and Immunity, College of Medicine & Public Health, Flinders University of South Australia, Bedford Park, SA 5042, Australia; 7 Department of Medicine, Brigham and Women’s Hospital, Boston, MA 02115, USA; 8 Department of Medicine, Harvard Medical School, Boston, MA 02115, USA

**Keywords:** non-nutritive sweeteners, glucose absorption, gut microbiota, antibiotics, GLP-1

## Abstract

**Background/Objective:** High habitual consumption of non-nutritive sweeteners (NNS) is linked to increased incident type 2 diabetes, with emerging clinical evidence that effects on gut microbiota may, in part, drive this risk. However, the precise contribution of the effects of NNS on gut microbiota to host glycemic responses remains unclear. **Methods:** Ten-week-old male C57BL/6 mice (N = 10 per group) were randomized to drinking water with or without combined NNS (sucralose 1.5 mg/mL plus acesulfame-K 2.5 mg/mL) and with or without antibiotics to deplete gut microbiota (ABX, 1 mg/mL ampicillin and neomycin) over two weeks. Oral glucose tolerance tests (OGTT, 2 g/kg) were conducted on days −1 and 12. On day 14, mice underwent a jejunal infusion of glucose (300 mg) with 3-O-methyl glucose (30 mg, 3-OMG, a marker of glucose absorption) in 1.5 mL for 30 min, followed by blood collection and bioassays. Data were analyzed using ANOVA with NNS and ABX as factors. **Results:** Jejunal glucose absorption was augmented in NNS+ mice relative to NNS− (31%; 3-OMG T30; *p* ≤ 0.05) independent of ABX. ABX attenuated OGTT responses independent of NNS supplementation (−35%; incremental AUC, *p* ≤ 0.001). NNS+ ABX+ mice had augmented GLP-1 responses to intrajejunal glucose relative to other groups (69–108%, *p* < 0.05). **Conclusions:** These findings demonstrate that sub-acute NNS supplementation augments glucose absorption independent of gut microbiota in mice but does not disrupt glycemic responses. Antibiotic depletion of gut microbiota markedly increased glucose tolerance in mice, which may involve the actions of GLP-1.

## 1. Introduction

Recent decades have seen a marked increase in the consumption of non-nutritive sweeteners (NNS) [1]. Despite being perceived and marketed as “healthier” than sugar, epidemiological studies have revealed that high habitual consumption of NNS-containing beverages is associated with an increased risk of developing type 2 diabetes (T2D) [2,3,4]. However, findings from interventional studies with NNS are equivocal [5,6], and the precise nature of this risk remains unclear.

Sweet taste receptors, heterodimers of two G-protein coupled receptors T1R2 and T1R3, recognize all known sweet tastants and are present on subsets of enteroendocrine cells within the gut [7,8]. The binding of sweet ligands to STRs can trigger the release of gut hormones including glucagon-like peptide-1 and -2 (GLP-1, GLP-2) from L-cells, and glucose-dependent insulinotropic polypeptide (GIP) from K-cells. GLP-1 and GIP augment postprandial glucose-dependent insulin secretion [9,10] while GLP-2, co-released with GLP-1, enhances the function of the primary apical glucose transporter sodium-glucose cotransporter-1 (SGLT-1), and potentially the basolateral glucose transporter 2 (GLUT2), to facilitate glucose uptake to the circulation [11,12]. While the NNSs sucralose and acesulfame-K, or their combination, do not alter oral glucose-evoked GLP-1 release acutely in humans [13], they are not metabolically inert, and can both trigger in vivo and ex vivo release of GLP-1 in rodents and human duodenum and ileum tissues respectively [14,15] and upregulate SGLT-1 and GLUT2 expression and function [16,17,18]. In this manner, habitual activation of intestinal STR by diets high in either carbohydrates or NNS has the potential to augment SGLT-1 capacity over time, thereby exacerbating postprandial glycemic excursions.

Gut microbiota are fundamental to metabolic homeostasis and critical in satiety signaling and glycemic control [19,20,21,22]. Mice with depleted gut microbiota, either as a result of antibiotic treatment or being raised in gnotobiotic (germ-free) conditions, display marked alterations in metabolic function [23,24,25], underscoring the critical link between prevailing diet, gut microbiota, and host metabolism [26,27,28,29,30,31,32]. Indeed, fecal transplantation from NNS-supplemented mice and human donors to germ-free recipients has been shown to induce glucose intolerance in recipients that parallels that of the donor, supporting a causal link between microbiota composition change and NNS-evoked dysglycemia [33].

It remains uncertain, however, as to what proportion of NNS-evoked dysglycemia is driven by effects on host intestinal STR-SGLT-1 mediated glucose absorption, versus effects on hosted gut microbiota that disrupt post-biotic signals and host glycemic responses. As such, we sought to determine the effects of an NNS combination, sucralose and acesulfame-K, commonly found in diet beverages, on glycemic control in C57BL/6 mice, and whether this differed in mice with depleted gut microbiota following antibiotic treatment.

## 2. Materials and Methods

### 2.1. Mice

Eight-week-old male C57BL/6 mice were bred and group-housed (maximum four mice/cage) in pathogen-free conditions at the South Australian Health and Medical Research Institute (SAHMRI) Bioresources Facility (Adelaide SA, Australia) under constant temperature (22 ± 0.5 °C) and humidity (40–60%) with *ad libitum* access to a standard chow diet (Teklad Global Diet #2918: 18.6% protein, 6.2% fat; Harlan IN, USA) and water. Mice were habituated to a reversed 12 h light-dark cycle with lights off (Zeitgeber time 12, ZT12) at 10:00 over two weeks prior to experimental use to investigate the intestinal STR-SGLT-1 axis during its circadian peak of dark phase ingestion. Mice were then randomized in groups of N = 10 to water with or without NNS (NNS+, NNS−, respectively) containing 1.5 mg/mL sucralose (Melbourne Food Depot, Melbourne Vic, Australia) plus 2.5 mg/mL acesulfame-K (Melbourne Food Depot, Melbourne Vic, Australia) over two weeks in the presence or absence of combined antibiotics (ABX+, ABX− respectively) (ampicillin 1 mg/mL plus neomycin 1 mg/mL; Sigma-Aldrich, Bayswater Vic, Australia), to broadly target gram-negative and gram-positive bacteria [34]. NNS doses were based on concentration-dependent NNS preference in C57BL/6 mice [35] and equated to one-tenth the dose used in previous five-day twice-daily oral gavage over experiments [36], which informed the two-week chronic exposure here. Mice were weighed daily, and water intake was monitored throughout the study. All experiments were approved by the SAHMRI Animal Ethics Committee (SAM 20.021) and conducted in compliance with the Australian code for the care and use of animals for scientific purposes and ARRIVE guidelines [37].

### 2.2. Oral Glucose Tolerance Tests (OGTT)

An oral glucose tolerance test (OGTT) was performed at ZT13, one day prior to commencing drinking water interventions (pre-study; day −1). Mice were fasted for three hours in wire-bottomed cages with *ad libitum* access to water, then gavaged with a glucose bolus (2 g/kg body weight); blood glucose was measured at baseline and at 10, 20, 30, 45, 60, 90 and 120 min using a glucometer (FreeStyle Optium Neo H, Abbott Diabetes Care, Alameda CA, USA,). OGTTs were repeated on day twelve in an identical manner (post-study; day 12). All OGTTs were performed under red-light, to maintain the light cycle phase.

### 2.3. Intestinal Glucose Infusion, Blood & Tissue Collection

Mice were fasted for three hours in wire-bottomed cages on day 14 and then anesthetized with 4% isoflurane in oxygen at ZT13. A midline laparotomy was performed, and an in-flow flexible cannula was inserted in the aboral direction into the proximal jejunum at the ligament of Treitz and secured with a 4-0 silk ligature (Ethicon, Johnson & Johnson Medical, North Rye, NSW Australia); an out-flow cannula was inserted 8-cm distally. A glucose solution (300 mg glucose + 30 mg 3-O-methyl-glucose, 3-OMG, in 1.5 mL) was infused via the proximal cannula using a syringe pump (Alaris GH Plus, Becton Dickinson, NJ, USA) at a rate of 3 mL/h for 30 min. Blood glucose concentrations were measured using a glucometer prior to infusion and at 10, 20, and 30 min of infusion before collecting terminal cardiac blood in K3EDTA collection tubes (MiniCollect^®^ 1 mL, Greiner Bio-One, Kremsmünster, Austria), which was centrifuged (4 °C at 3200× *g*, 10 min) and stored at −80 °C. Duodenum, jejunum, and terminal ileum were removed, and the lumen was flushed with phosphate buffer at 4 °C; a portion of each section was cut longitudinally, flattened, and scraped to collect mucosa, which was snap-frozen in liquid N_2_ and stored at −80 °C ahead of molecular analyses.

### 2.4. Glucose Absorption and Gut Hormone Analyses

Plasma concentrations of the non-metabolizable glucose analog 3-OMG were measured as an index of glucose absorption using commercial high-performance liquid chromatography-mass spectrometry (Agilex Biolabs, Thebarton, SA, Australia) with a sensitivity of 10 pmol/L. Plasma GIP, total GLP-1, glucagon, insulin, peptide YY (PYY), and pancreatic polypeptide (PP) concentrations were measured using a multi-hormone MILLIPLEX Mouse Metabolic Hormone Expanded Panel (MPMMHE44K; Merck Millipore, Abacus dx, Meadowbrook, QLD, Australia). Sensitivity and assay coefficients of variation are shown in Supplementary Table S1. The glucagon-to-insulin ratio was calculated as a surrogate of endogenous glucose production [38].

### 2.5. Gene Expression Analysis

Mucosal RNA was extracted using a PureLink RNA Mini Kit (Invitrogen, Thermo Fisher Scientific, Adelaide Airport, SA, Australia) with RNA quantity and quality measured using a NanoDrop Lite Spectrophotometer (Thermo Fisher Scientific).

Region-specific expression of *Tas1r2* (T1R2), *Slc5a1* (SGLT-1), *Slc2a2* (GLUT2), *Gcg* (preproglucagon), *Pcsk1* (prohormone convertase 1), *Pcsk2* (prohormone convertase 2), and *Dpp4* (dipeptidyl peptidase IV, DPP IV) was determined for duodenum, jejunum and terminal ileum relative to the geometric mean of *B2m* (β2-microglobulin) and *Hprt* (hypoxanthine phospho-ribosyltransferase 1) using two-step quantitative real-time PCR (qRT-PCR, QuantiTect SYBR Green PCR kit, Qiagen, Hilden, Germany) and bioinformatically validated primers (Supplementary Table S2). Briefly, qRT-PCR was performed for 42 cycles on a thermocycler (Applied Biosystems QuantStudio 7, Thermo Fisher Scientific) with a melt curve generated (60–95 °C) to verify product specificity and identity; each assay was performed in triplicate and included no-template and no-reverse transcriptase controls. Expression, relative to the housekeeper geometric mean, was averaged for each target at 10 ng of total RNA.

### 2.6. Fecal Collection, DNA Extraction and Bioinformatic Analysis

Fecal pellets passed on days 0 and 14 were collected from individual mice with sterile forceps and placed into sterile 1.5 mL Eppendorf tubes and stored at −80 °C. Bacterial DNA was extracted using the DNeasy PowerSoil kit (Qiagen) as previously described [39], and qRT-PCR was used to determine the abundance of the 16S rRNA gene to determine total bacterial load [39,40]. The V4 hypervariable region was used to generate and index amplicon libraries due to its high taxonomic resolution, universal primers, and validated bioinformatic tools, as previously described [39]. These libraries were sequenced using a Miseq Reagent Kit v3 (2 × 300 bp) on an Illumina Miseq platform (Illumina, Scoresby, VIC, Australia) at the South Australian Genomics Centre (SAHMRI, Adelaide, SA, Australia). Bioinformatic analysis of paired-end sequence reads was performed using QIIME2 software (v2021.11) [41]; sequences were de-multiplexed, quality-filtered (based on scores above Q30) and de-noised using Dada2 [42]. Taxonomic classification of amplicon sequence variants was performed against the SILVA138 16S rRNA reference database clustered at 99% similarity. All samples were subsampled to 4372 sequence reads.

### 2.7. Statistical Analysis

Glycemic responses were analyzed using three-way repeated measures ANOVA with NNS, ABX, and time as factors. Incremental areas under the curve (iAUC) for blood glucose responses were calculated using the trapezoidal rule and analyzed using two-way ANOVA with NNS and ABX as factors. Fasting blood glucose, plasma 3-OMG and hormone concentrations, and gene expression data were analyzed using two-way ANOVA with NNS and ABX as factors. Post hoc comparisons with adjusted *p* values for multiple comparisons used Bonferroni’s correction when ANOVAs showed significant interactions. Within-group changes in body weight, total bacterial abundance, and measures of alpha diversity were analyzed using Wilcoxon tests. Between-group post-study differences were analyzed using two-way ANOVA with NNS and ABX as factors. All data are expressed as mean ± SEM. All analyses were conducted using SAS (v9.4M6, Cary, NC, USA) or Prism (v9.0.0 Graphpad, La Jolla, CA, USA).

## 3. Results

### 3.1. Mice

**Figure 1:**
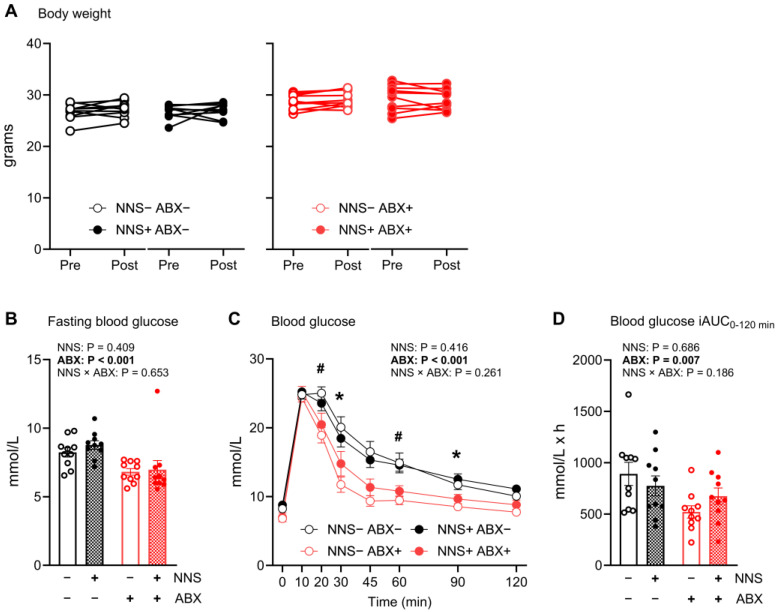
Body weight, fasting blood glucose, and oral glucose tolerance test responses (day 12). (**A**) Pre- and post-study body weight (**B**) fasting blood glucose concentrations (day 12), (**C**) OGTT blood glucose responses, and (**D**) iAUC_0–120_. *p* values above for main and interaction effects with bold signifying significance; * *p* < 0.05 and ^#^ *p* < 0.01 in mixed models (ABX × time). N = 10. Data are mean ± SEM. ABX, antibiotic-supplemented; iAUC, incremental area under the curve; NNS, non-nutritive sweetener-supplemented; OGTT, oral glucose tolerance test.

There was no significant difference in pre- and post-study body weight between any groups (Figure 1A). Group cages showed heterogeneity for water intake in NNS and ABX-treated groups (Supplementary Figure S1).

### 3.2. Oral Glucose Tolerance Tests

All groups had similar pre-study (day −1) fasting blood glucose concentrations and OGTT responses (iAUC, Supplementary Figure S2A–C). Day 12 fasting blood glucose concentrations (Figure 1B) and OGTT responses (Figure 1C,D) did not differ between NNS+ and NNS− mice. Day 12 fasting blood glucose concentrations were 21% lower in ABX+ compared to ABX− mice (*p* < 0.001, Figure 1B) while OGTT responses were 35% lower (iAUC; *p* = 0.007, Figure 1D) with significant differences at T = 20, 30, 60, 90 min (all *p* < 0.05, Figure 1C).

### 3.3. Glycemic Responses and Glucose Absorption Following Intrajejunal Glucose Infusion (Day 14)

**Figure 2:**
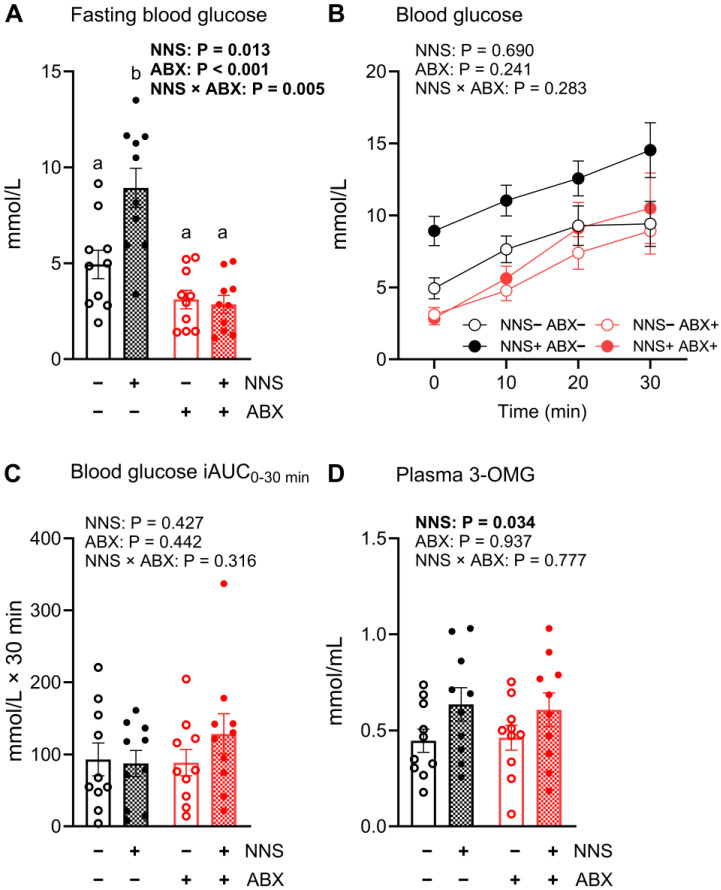
Fasting blood glucose concentrations and glycemic responses to intrajejunal glucose infusion (day 14). (**A**) Fasting blood glucose concentrations (day 14), (**B**) blood glucose responses, (**C**) iAUC0 30, and (**D**) plasma 3-OMG concentrations as a marker of glucose absorption following intrajejunal infusion of glucose (300 mg) and 3-OMG (30 mg). *p* values above for main and interaction effects with bold signifying significance; a, b denote interaction differences (post hoc multiple comparison) at *p* < 0.05. N = 10. Data are mean ± SEM. 3-OMG, 3-O-methyl glucose; ABX, antibiotic-supplemented; iAUC, incremental area under the curve; NNS, non-nutritive sweetener-supplemented; OGTT, oral glucose tolerance test.

Day 14 fasting blood glucose concentrations were 38% higher in NNS+ compared to NNS− mice (*p* = 0.013), and 80% lower in ABX+ compared to ABX− mice (*p* < 0.001). A significant ABX × NNS interaction (*p* = 0.005) was also evident, with fasting blood glucose concentrations 58–103% higher in NNS+ ABX− mice compared to all other groups (all *p* < 0.05, Figure 2A). There were no differences in glycemic responses to intrajejunal glucose infusion between any groups following baseline adjustment (Figure 2B,C). Plasma 3-OMG concentrations were 31% higher in NNS+ compared to NNS− mice after the 30 min glucose infusion, independent of ABX (*p* = 0.034, Figure 2D); plasma 3-OMG concentrations did not differ between ABX+ and ABX− mice.

### 3.4. Hormone Responses

**Figure 3:**
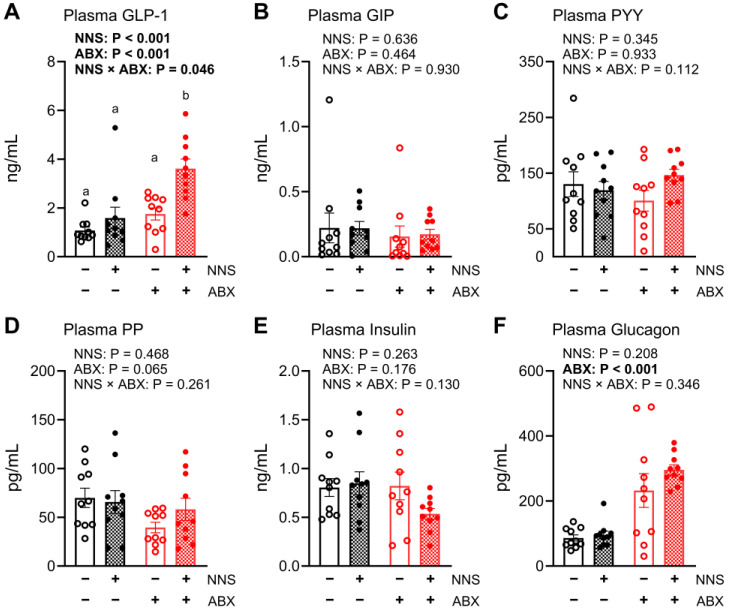
Plasma hormone responses to intrajejunal glucose infusion. (**A**) Plasma total GLP-1, (**B**) GIP, (**C**) PYY, (**D**) PP, (**E**) insulin, and (**F**) glucagon (**F**) concentrations after 30 min intrajejunal glucose infusion. *p* values above for main and interaction effects with bold signifying significance; a, b denote interaction differences (post hoc multiple comparison) at *p* < 0.05. N = 10. ABX, antibiotic-supplemented; GIP, glucose-dependent insulinotropic polypeptide; GLP-1, glucagon-like peptide-1; NNS, non-nutritive sweetener-supplemented; PP, pancreatic polypeptide; PYY, peptide YY.

Plasma total GLP-1 concentrations were 59% higher in NNS+ compared to NNS− mice (*p* < 0.001) and 68% higher in ABX+ compared to ABX− mice (*p* < 0.001). An NNS × ABX interaction was evident for total GLP-1 (*p* = 0.046), with total GLP-1 concentrations 69–108% higher in NNS+ ABX+ mice after intrajejunal glucose infusion compared to all other groups (all *p* < 0.05, Figure 3A). Plasma glucagon concentrations were twice as high in ABX+ compared to ABX− mice (*p* < 0.001, Figure 3F). In contrast, GIP, PYY, PP, and insulin concentrations did not differ between any groups (Figure 3B–E). The glucagon-to-insulin ratio was 64% higher in NNS+ compared to NNS− mice (*p* = 0.009) and 116% higher in ABX+ compared to ABX− mice (*p* < 0.001). An NNS × ABX interaction (*p* = 0.016) was also evident for the glucagon-to-insulin ratio, which was 77–140% higher in NNS+ ABX+ mice compared to all other groups (all *p* < 0.05, Supplementary Figure S3).

### 3.5. Intestinal Gene Expression

#### 3.5.1. Duodenum (Not Exposed to Glucose Infusion)

**Figure 4:**
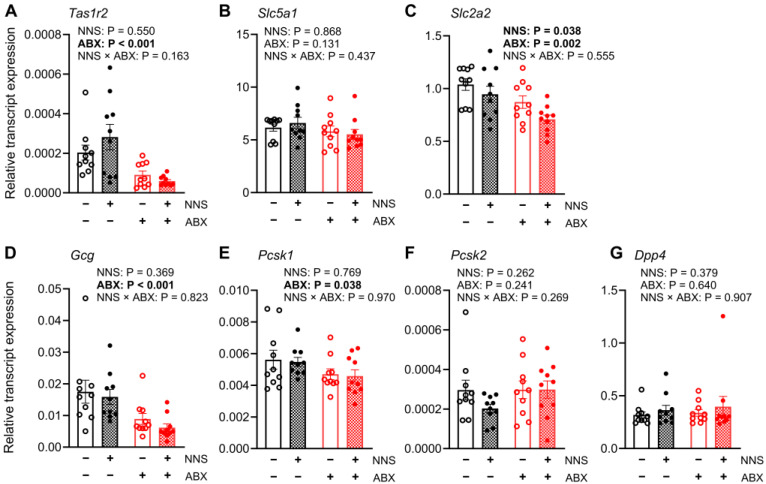
Expression of duodenal transcripts. Relative expression of (**A**) *Tas1r2*, (**B**) *Slc5a1*, (**C**) *Slc2a2* (**D**) *Gcg*, (**E**) *Pcsk1*, (**F**) *Pcsk2*, and (**G**) *Dpp4* transcripts in duodenal mucosa collected after 30 min jejunal glucose infusion. *p* values above for main and interaction effects with bold signifying significance. N = 10. ABX, antibiotic-supplemented; NNS, non-nutritive sweetener-supplemented.

Compared to NNS− mice, NNS+ mice had a lower relative duodenal expression of *Slc2a2* transcripts (−14%, *p* = 0.038, Figure 4C), while expression of other transcripts was similar. Compared to ABX− mice, ABX+ mice had lower relative duodenal expression of *Tas1r2* (−105%; *p* < 0.001, Figure 4A), *Slc2a2* (−23%; *p* = 0.002, Figure 4C), *Gcg* (−75%; *p* < 0.001, Figure 4D) and *Pcsk1* transcripts (−18%; *p* = 0.038, Figure 4E). The relative duodenal expression of *Slc5a1*, *Pcsk2*, and *Dpp4* transcripts did not differ between any groups (Table 1).

#### 3.5.2. Jejunum (Exposed to Glucose Infusion)

**Figure 5:**
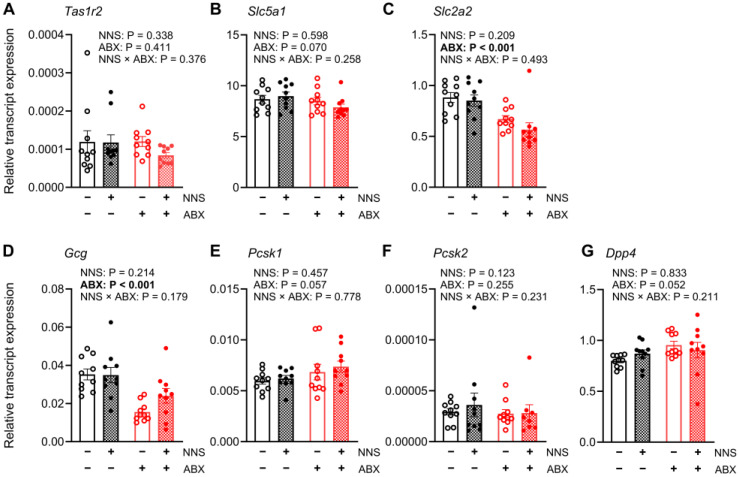
Expression of jejunal transcripts. Relative expression of (**A**) *Tas1r2*, (**B**) *Slc5a1*, (**C**) *Slc2a2* (**D**) *Gcg*, (**E**) *Pcsk1*, (**F**) *Pcsk2*, and (**G**) *Dpp4* transcripts in jejunal mucosa collected after 30 min glucose infusion. *p* values above for main and interaction effects with bold signifying significance. N = 10. ABX, antibiotic-supplemented; NNS, non-nutritive sweetener-supplemented.

NNS+ and NNS− mice had similar relative jejunal expression of all transcripts (Figure 5). Compared to ABX− mice, ABX+ mice had lower relative jejunal expression of *Slc2a2* (−34%; *p* < 0.001, Figure 5C) and *Gcg* transcripts (−56%; *p* < 0.001, Figure 5D) and trends for lower expression of *Slc5a1* (−8%; *p* = 0.070, Figure 5B) and *Dpp4* transcripts (−11%; *p* = 0.052, Figure 5F), and higher expression of *Pcsk1* transcript (15%; *p* = 0.057, Figure 5E). The relative expression of jejunal *Tas1r2* and *Dpp4* transcripts did not differ between any groups (Table 1).

#### 3.5.3. Terminal Ileum (Not Exposed to Glucose Infusion)

**Figure 6:**
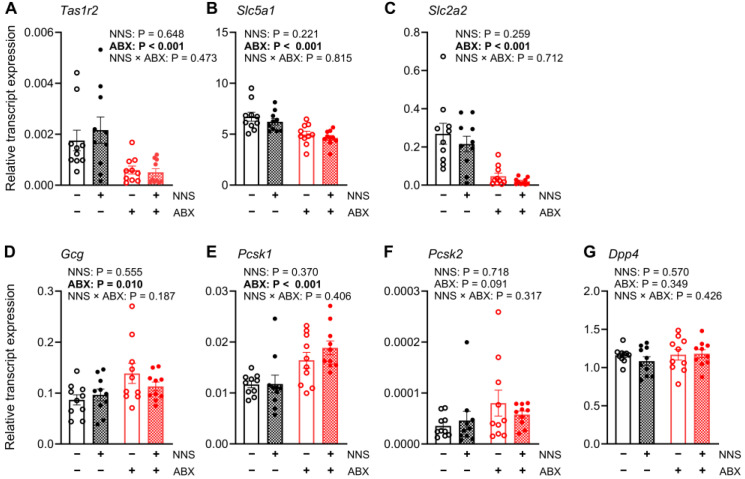
Expression of ileal transcripts. Relative expression of (**A**) *Tas1r2*, (**B**) *Slc5a1*, (**C**) *Slc2a2* (**D**) *Gcg*, (**E**) *Pcsk1*, (**F**) *Pcsk2*, and (**G**) *Dpp4* transcripts in ileal mucosa collected after 30 min jejunal glucose infusion. *p* values above for main and interaction effects with bold signifying significance. N = 10. ABX, antibiotic-supplemented; NNS, non-nutritive sweetener-supplemented.

NNS+ and NNS− mice had similar relative ileal expression of all transcripts (Figure 6, Table 1). Compared to ABX− mice, ABX+ mice had lower relative ileal expression of *Tas1r2* (−114%; *p* < 0.001, Figure 6A), *Slc5a1* (−28%; *p* < 0.001, Figure 6B) and *Slc2a2* transcripts (−152%; *p* < 0.001, Figure 6C), and higher relative ileal expression of *Gcg* (34%; *p* = 0.010, Figure 6D) and *Pcsk1* transcripts (47%; *p* < 0.001, Figure 6E). The relative ileal expression of *Pcsk2* and *Dpp4* transcripts did not differ between any groups (Table 1).

### 3.6. Fecal Bacterial Load, Microbiota Diversity, Composition in ABX− Mice

**Figure 7:**
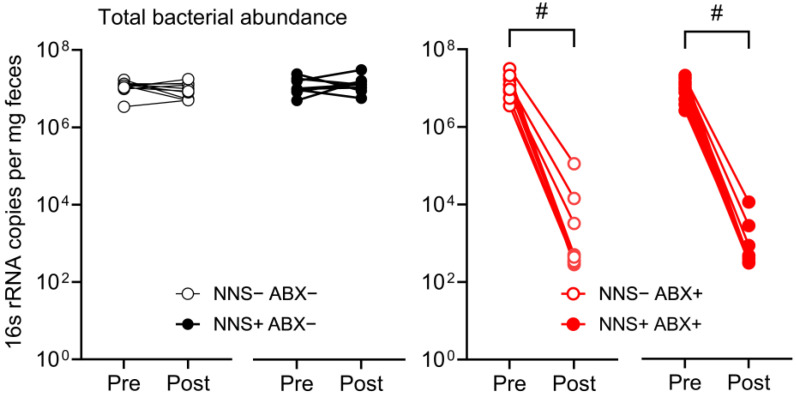
Total bacterial abundance pre- and post-study. Total bacterial abundance assessed via 16s rRNA gene copies per mg feces measured pre- and post-study in ABX− (**left**) and ABX+ (**right**) cohorts. # *p* < 0.01, N = 10. ABX, antibiotic-supplemented; NNS, non-nutritive sweetener-supplemented.

Post-study total bacterial abundance was unaltered in ABX− mice but was significantly reduced in ABX+ mice relative to pre-study abundance (99.9% decrease *p* = 0.002, Figure 7).

**Figure 8:**
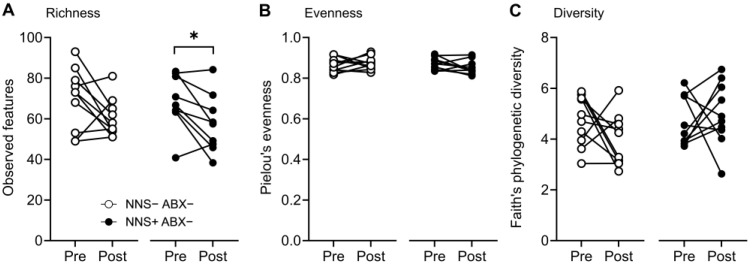
Alpha diversity measures of fecal microbiome richness, evenness, and diversity. (**A**) Pre- and post-study alpha diversity measures of richness (observed features), (**B**) evenness (Pielou’s Evenness index), and (**C**) diversity (Faith’s phylogenetic diversity index) in NNS ± ABX− mice. * *p* < 0.05, N = 10. ABX, antibiotic-supplemented; NNS, non-nutritive sweetener-supplemented.

NNS+ mice had reduced fecal microbiota alpha diversity measures of richness (observed species; −17%; *p* = 0.016) on day 14 compared to day 0, while richness was unchanged in NNS− mice. Alpha diversity measures of evenness (Pielou’s) and diversity (Faith’s phylogenetic diversity) were unchanged in both NNS− and NNS+ mice (Figure 8).

Pre- and post-study microbiota composition was similar in ABX− mice as determined by Bray Curtis distances (Table 2). There were no significant paired changes observed between pre- and post-study time points in NNS+ (*p* = 0.242) or NNS− (*p* = 0.446) mice when adjusted for potential cage effects. Paired comparisons between pre- and post-study time points also indicated that the relative abundance of individual taxa remained unchanged in NNS and NNS− mice.

## 4. Discussion

This study determined the effects of two-week supplementation with a combination of non-nutritive sweeteners (NNS), sucralose, and acesulfame-K, on glucose handling, gut hormones, and intestinal transcription in C57BL/6 mice in the context of an intact or antibiotic-depleted gut microbiota. These NNS augmented jejunal glucose absorption independent of gut microbiota depletion and augmented GLP 1 responses to jejunal glucose in a partly microbiota-mediated manner but did not alter oral glucose tolerance. Depletion of gut microbiota, using broad-spectrum antibiotics ampicillin and neomycin (ABX), lowered fasting blood glucose over two weeks and improved glucose tolerance independent of NNS supplementation. ABX also augmented plasma GLP 1 and glucagon responses to intrajejunal glucose infusion, increased ileal expression of *Gcg* and *Pcsk1* transcripts, and reduced *Slc2a2* in all intestinal regions. We conclude that sub-acute NNS supplementation augments jejunal glucose uptake in C57BL/6 mice independent of gut microbiota without changing glucose tolerance. The improved glucose tolerance due to gut microbiota depletion extends existing evidence of this effect [23,24,33,43] and may be mediated by augmented release of ileal GLP-1 and increased glucose disposal in the intestinal epithelium.

Sub-acute sucralose and acesulfame-K supplementation exerted a profound effect to augment jejunal glucose absorption in C57BL/6 mice, of similar magnitude but opposite direction to the effects of metformin on glucose absorption in rodents [44,45] and healthy humans [46,47]. That oral glucose tolerance and blood glucose responses to jejunal glucose infusion were unaffected by NNS indicates an ability to compensate for the change in glucose handling, at least in the short term, potentially through changes in insulin sensitivity. The lack of change in glucose tolerance contrasts studies with longer drinking water exposure to sucralose where dose-dependent increases in duodenal glucose absorptive capacity and glucose intolerance were apparent after 11–12 weeks in C57BL/6 mice [33,48]. It also contrasts glucose intolerance seen after 11 weeks of saccharin exposure in the context of a high-fat diet [33]. As such, NNS may drive glucose intolerance with a longer duration of exposure in mice, or if there is an additional metabolic stressor at play. The increased glucose tolerance seen in ABX+ mice, discussed below, is likely to have provided a further defense against developing dysglycemia by buffering against enhanced intestinal glucose absorption in NNS+ ABX+ mice.

Interestingly, the effect of NNS to augment glucose absorption upon jejunal infusion occurred without any change in jejunal transcription of *Tas1r2*, or glucose transporters *Slc5a1* or *Slc2a2* due to NNS. These findings contrast our previous work where twice-daily sucralose gavage augmented jejunal *Slc5a1* transcript expression in C57BL/6 mice on a standard chow diet over four days [36]. Moreover, two-week supplementation with sucralose or acesulfame K in drinking water in C57BL/6 mice on an ultra-low-carbohydrate diet increased expression of small intestinal *Slc5a1* transcripts two-fold, and in mice supplemented with sucralose, a corresponding increase in SGLT-1 protein and glucose uptake, effects that were absent in mice deficient in sweet taste molecules T1R3 and G-protein α-gustducin [49]. This infers that NNS effects on the intestinal STR-SGLT-1 pathway in mice are confounded by the form and dose of NNS exposure, as well as by the high carbohydrate content (60%) of a standard chow diet.

Evidence from preclinical and clinical studies supports an emerging and causal role of NNS-mediated alteration in gut microbiota as a driver of glucose intolerance [33,50,51]. Despite a reduction in the number of unique bacterial taxa, overall fecal microbiota composition in NNS+ ABX− mice here did not change significantly. However, NNS effects to augment glucose absorption occurred independent of gut microbiota, supporting a direct host-mediated effect. In contrast, the powerful NNS+ ABX+ interaction effect on glucose-evoked GLP-1 concentrations suggests that NNS effects on GLP 1 release are mediated by both the host and microbiota (hosted) and that NNS augment glucose absorption and GLP-1 release via distinct mechanisms. Despite a marked increase in glucose-evoked total GLP-1 responses in NNS+ mice, transcripts related to intestinal active GLP-1 production (preproglucagon (*Gcg*) and prohormone convertase-1 (*Pcsk1*)), or GLP-1 catabolism (dipeptidyl peptidase 4 (*Dpp4*)) in the glucose-infused jejunum were unaffected.

The effects of ABX to lower fasting blood glucose and increase glucose tolerance that we observed here accord with extensive literature in antibiotic-depleted and germ-free mice [23,24,33,43]. When resolving these glycemic responses to ten-minute time intervals, it is apparent that the rise to peak blood glucose at ten minutes is identical in ABX+ and ABX− mice, before resolving more rapidly in ABX+ mice beyond this time. This may relate to augmented GLP-1 release after initial gastric emptying in ABX+ compared to ABX− mice, as has been reported previously [23]. Indeed, the timing of blood glucose resolution in our OGTTs is consistent with an action of GLP 1 to slow gastric emptying [52]; such a resolution was not evident in the jejunal infusion experiments where gastric emptying was bypassed.

Augmented total GLP-1 and glucagon responses to jejunal glucose infusion in ABX+ mice coincided with lower transcript expression of jejunal *Slc2a2* and *Gcg*, without significant changes in other jejunal transcripts. This may relate to the fact that the transcriptional status of the glucose-infused jejunum differed from the non-infused duodenum and ileum. Indeed, ABX supplementation markedly lowered *Tas1r2* expression in the non-infused duodenum and ileum but not jejunum, where glucose exposure is likely to have upregulated *Tas1r2*, as we observed in healthy humans [53]. The expression of glucagon-producing prohormone convertase 2 (*Pcsk2*) and *Dpp4* transcripts did not change in any intestinal region in ABX+ mice. However, *Gcg* and *Pcsk1* were specifically increased by ABX in the ileum, the region of peak expression for both transcripts. If this extends to proteins, it infers a preferential increase in active GLP 1 production capacity in the large ileal L-cell pool in ABX+ mice. As microbiota-derived SCFAs serve as energy substrates for the intestinal epithelium, and microbiota depletion reduces SCFA availability [24,54], we speculate that such an ABX-dependent increase in GLP-1 would slow intestinal transit to safeguard the provision of intestinal energy [23]. Finally, the effects of ABX to lower transcript expression of *Tas1r2* in non-infused duodenum, and *Slc2a2* in all regions, may collectively limit STR-dependent gains in proximal glucose absorption to increase nutrient access to the more distal gut.

The ABX-dependent attenuation of *Slc2a2* expression in all intestinal regions, with distal predominance, supports the concept that gut microbiota depletion causes enterocytes to limit glucose efflux [23]. However, there was no corresponding lowering of jejunal glucose absorption, potentially due to a disconnect between transcript expression and protein function or the timeline of de novo GLUT2 translocation. A concomitant increase in GLP-2-mediated intestinotrophic effects in ABX+ mice could also facilitate epithelial proliferation and increase energy demand [55,56]. Indeed, disruption of the gut microbiota by four-week treatment with ampicillin, vancomycin, and neomycin in drinking water increased jejunal villous height and surface area markedly in C57BL/6 mice [57]. This change in intestinal energy demand is likely to alter whole-body glucose homeostasis [23]. The increased glucagon-to-insulin ratio in NNS+ ABX+ mice suggests endogenous glucose production is augmented as a countermeasure to augmented GLP-1 release, as has been shown in antibiotic-treated C57BL/6 mice [23] and demands that future research routinely measure fasting glucagon and hepatic gluconeogenesis in mice. While direct effects of ABX on the host must also be considered, similarities in glucose tolerance between antibiotic depletion of gut microbiota here and by others [23,33,43], as well as germ-free mice [24], support the concept that ABX effects are largely due to gut microbiota depletion.

We acknowledge study limitations, including the relatively brief NNS exposure of two weeks. Differences in water intake between groups also have the potential to influence blood volume, ion exchange within the intestinal lumen, gut microbiota, and renal function, which may affect study outcomes. We used male mice in this proof-of-concept study only and acknowledge the importance of including female mice in future investigations. We did not measure food intake or body composition and are thus unable to report on any potential differences in energy intake. Due to the blood volume of mice, we were unable to measure plasma hormone and 3-OMG concentrations during the OGTT, or longitudinally during the jejunal glucose infusion. We used 3-OMG as our primary measure of glucose absorption but acknowledge that intestinal transcript expression did not change despite NNS augmentation of glucose absorption, and we did not measure protein expression. This is particularly important for SGLT-1, where cAMP-dependent post-transcriptional stabilization is the major mode of protein upregulation [12] and the likely reason *Slc5a1* transcript expression did not change here. The use of anesthesia during jejunal glucose infusions, but not during OGTTs, is a likely confounder in the observed NNS effect of augmented fasting blood glucose in the former. Finally, gut bacteria were profiled using 16S rRNA amplicon sequencing, which limits the identification of bacterial amplicon sequence variants to the genus level.

## 5. Conclusions

In conclusion, we have shown that two-week supplementation with an NNS combination frequently used in the food industry (sucralose and acesulfame K) augments jejunal glucose absorption independent of antibiotic depletion of gut microbiota, in support of a direct effect of these NNS on the host. Despite this, glucose tolerance and glycemic responses to jejunal glucose infusion did not change over the sub-acute duration of this NNS supplementation. Our observations also support the concept that antibiotic depletion of gut microbiota increases glucose tolerance in mice and provides a mechanistic basis via augmented GLP 1 and glucagon release, and potentially, GLP 1 dependent slowing of gastric emptying and intestinal transit, together with augmented glucose disposal within the intestinal epithelium. While it is not feasible to administer antibiotics to people with T2D to leverage such benefits, the identification of specific gut bacteria that mediate or moderate antibiotic-dependent increases in GLP-1 may have substantial utility in future drug development.

## Figures and Tables

**Table 1:** Summary of transcript expression differences.

	*Tas1R2*		*Slc5a1*		*Slc2a2*		*Gcg*		*Pcsk1*		*Pcsk2*		*Dpp4*	
	NNS	ABX	NNS	ABX	NNS	ABX	NNS	ABX	NNS	ABX	NNS	ABX	NNS	ABX
Duodenum	NC	▼	NC	NC	▼	▼	NC	▼	NC	▼	NC	NC	NC	NC
* Jejunum	NC	NC	NC	NC	NC	▼	NC	▼	NC	NC	NC	NC	NC	NC
Ileum	NC	▼	NC	▼	NC	▼	NC	▲	NC	▲	NC	NC	NC	NC

Note: Arrows denote the modulation (red—decrease, green—increase) of relative transcript expression compared to respective controls (NNS− and ABX−). * Exposed to glucose infusion. ABX, antibiotic-supplemented; NC, no change; NNS, non-nutritive sweetener-supplemented.

**Table 2:** Fecal microbiota composition changes with or without NNS.

Bray Curtis Distance	Pseudo-F	*p* (perm)	Unique Perms
Time	1.679	0.155	9947
NNS	0.774	0.640	9932
Time × NNS	0.638	0.704	9929
Cage (NNS)	1.442	0.056	9871
Mice (Cage, NNS)	1.863	0.020	9864

## Data Availability

All raw sequence data are publicly available from the NCBI Sequence Read Archive repository under BioProject PRJNA1133889. All other data will be made available upon request.

## Supplementary Materials

The following supporting information can be downloaded at https://www.mdpi.com/article/10.3390/nu16213628/s1, Figure S1: Daily drinking water intake; Figure S2: Pre-study fasting blood glucose concentrations and oral glucose tolerance (Day −1); Figure S3: Plasma glucagon-to-insulin ratio in response to intrajejunal glucose infusion; Table S1: Primers used in qRT-PCR assays; Table S2: Hormone assay sensitivity.
