# Supplementary material for: Host or the Hosted? Effects of Non-Nutritive Sweeteners on Intestinal and Microbial Mechanisms of Glycemic Control
Source: Nutrients. 2024 Oct 25;16(21):3628. doi: 10.3390/nu16213628 (PMC11548007; doi:10.3390/nu16213628)
Supplement: Supplementary file 1 [file nutrients-16-03628-s001.zip › nutrients-3265470-supplementary.pdf]

SUPPLEMENTARY MATERIAL

Host or the hosted? Effects of non-nutritive sweeteners on intestinal and microbial mechanisms of glycemic control

Braden D. Rose[1,2,3,4], Nektaria Pezos[1,2,4], Jocelyn M. Choo[5,6], Tongzhi Wu[2,3], Geraint B. Rogers[5,6], Kerry L. Ivey[7,8], Christopher K. Rayner[2,3], Richard L. Young[1,2,3,4]†

[1]Intestinal Sensing Group, [2]Adelaide Medical School and [3]Centre of Research Excellence in Translating Nutritional Science to Good Health, The University of Adelaide, Adelaide, SA 5005, Australia

[4]Diabetes, Nutrition & Gut Health and [5]Precision Medicine, Lifelong Health, South Australian Health & Medical Research Institute (SAHMRI), Adelaide, SA 5000, Australia

[5]Microbiome and Host Health, Lifelong Health, South Australia Health and Medical Research Institute, Adelaide, South Australia, Australia.[6]Infection and Immunity, College of Medicine & Public Health, Flinders University of South Australia, Bedford Park, SA 5042, Australia.

[7]Department of Medicine, Brigham and Women's Hospital, Boston, MA 02115, USA

[8]Department of Medicine, Harvard Medical School, Boston, MA 02115, USA

Corresponding Author†

Richard L Young PhD

Nutrition, Diabetes & Gut Health, Lifelong Health, South Australian Health & Medical Research Institute (SAHMRI)

PO Box 11060 Adelaide SA 5001, AUSTRALIA

Tel: +61 8 8128 4845; Email: richard.young@adelaide.edu.au

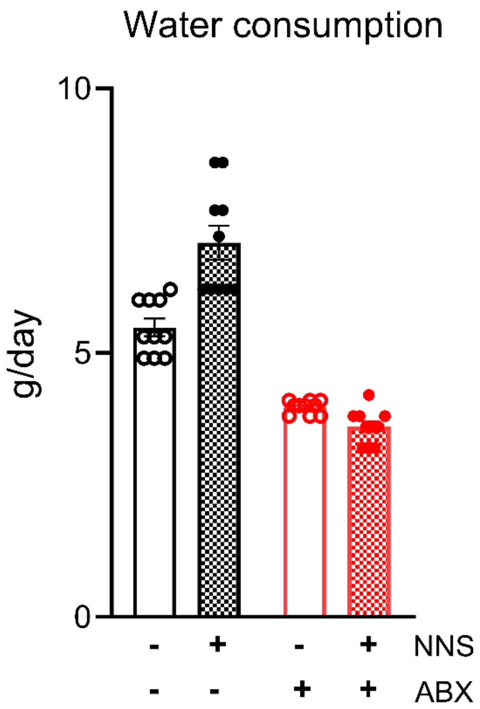

Supplementary Figure 1: Daily drinking water intake.

Daily drinking water intake in study cohorts. N = 10. ABX, antibiotic-supplemented; NNS, non-nutritive sweetener-supplemented.

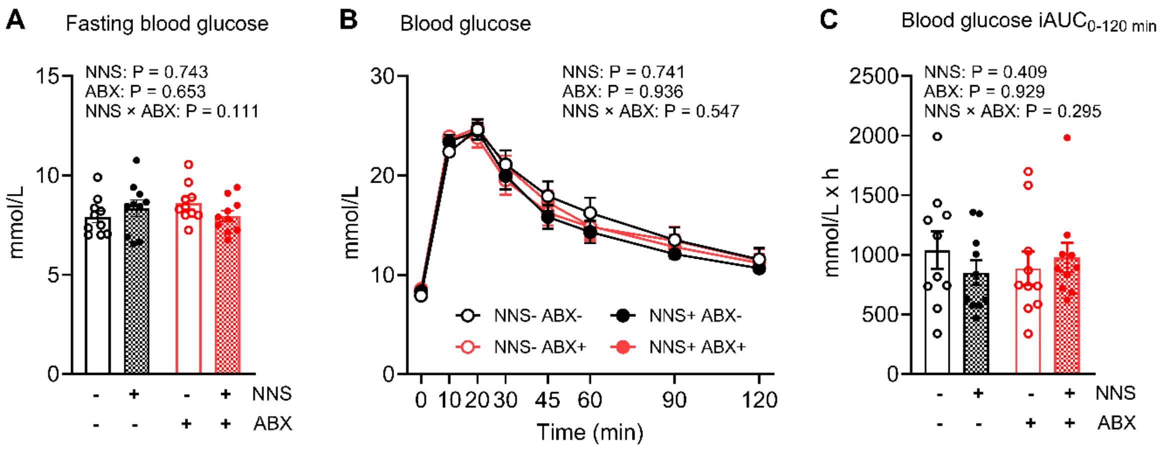

Supplementary Figure 2: Pre-study fasting blood glucose concentrations and oral glucose tolerance (Day -1).

(A) Fasting blood glucose concentrations (Day -1), (B) OGTT blood glucose responses, and (C) $iAUC_{0-120}$. P values above for main and interaction effects with bold signifying significance. N=10. Data are mean ± SEM. ABX, antibiotic-supplemented; iAUC, incremental area under the curve; NNS, non-nutritive sweetener-supplemented; OGTT, oral glucose tolerance test.

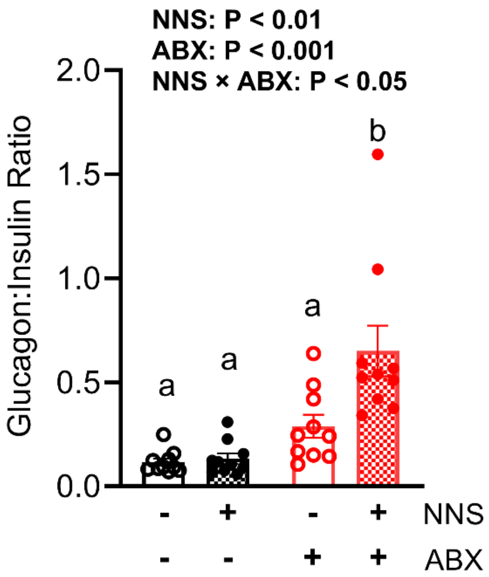

Supplementary Figure 3: Plasma glucagon-to-insulin ratio in response to intrajejunal glucose infusion.

Plasma glucagon-to-insulin ratio as an estimate of endogenous glucose production after 30-min intrajejunal glucose infusion. P values above for main and interaction effects with bold signifying significance; a, b denote interaction differences (post-hoc multiple comparison) at $P < 0.05$. N = 10. ABX, antibiotic-supplemented; NNS, non-nutritive sweetener-supplemented.

Supplementary Table 1: Hormone assay sensitivity

Analyte	Source (Catalogue No.)	Sensitivity (pg/ml)	CV %
**GLP-1 (total)**	Merck Millipore (MPMMMHE-44K)	22.8	3.6
**Glucagon**	Merck Millipore (MPMMMHE-44K)	14.4	1.7
**GIP**	Merck Millipore (MPMMMHE-44K)	1.4	4.4
**Insulin**	Merck Millipore (MPMMMHE-44K)	62.4	12.3
**PYY**	Merck Millipore (MPMMMHE-44K)	9.5	1.9
**PP**	Merck Millipore (MPMMMHE-44K)	6.1	1.8

Supplementary Table 2: Primers used in qRT-PCR assays

Gene (Protein)	QuantiTect Primer Assay (Locus, Qiagen Cat No.)	Amplicon length (bp)
***Tas1R2* (T1R2)**	**Mm_Tas1r2_1_SG** (NM_031873, XM_980714; QT00142639)	137
***Slc5a1* (SGLT-1)**	**Mm_Slc5a1_1_SG** (NM_019810; QT00112679)	120
***Slc2a2* (GLUT2)**	**Mm_Slc2a2_1_SG** (NM_031197; QT00103537)	105
***Gcg* (Proglucagon)**	**Mm_Gcg_1_SG** (NM_008100, XM_006498770; QT00124033)	94
***Pcsk1* (PC1)**	**Mm_Pcsk1_1_SG** (NM_013628, XM_006517153, XM_006517155, XM_006517154; QT00106190)	100
***Pcsk2* (PC2)**	**Mm_Pcsk2_1_SG** (NM_008792; QT00167426)	118
***Dpp4* (DPP-IV)**	**Mm_Dpp4_1_SG** (NM_001159543, NM_010074, XM_006498691, XM_006498690, XM_006498692; QT00095284)	70
***B2m* (β2M)**	**Mm_B2m_2_SG** (NM_009735; QT01149547)	143
***Hprt* (HPRT1)**	**Mm_Hprt_1_SG** (NM_013556; QT00166768)	168
